# Supplementary material for: Estimated Population Prevalence of Heart Failure with Reduced Ejection Fraction in Spain, According to DAPA-HF Study Criteria
Source: J Clin Med. 2020 Jul 3;9(7):2089. doi: 10.3390/jcm9072089 (PMC7408645; doi:10.3390/jcm9072089)
Supplement: Supplementary file 1 [file jcm-09-02089-s001.pdf]

Supplementary materials
Supplementary Tables

Table 1. Number of patients with the four selection criteria of the DAPA-HF study in the REDINSCOR cohort in Spain.

		Number of patients				
**Sex and age**	**N REDINSCOR**	**1 NYHA II-IV**	**2 EF ≤ 40%**	**3 GFR > 30**	**1+2+3 Diabetes**	**1+2+3 No diabetes**
**Men**						
35-44 years	117	117	101	115	14	85
45-54 years	259	259	220	256	65	152
55-64 years	486	486	412	475	168	233
65-74 years	472	472	390	446	186	183
75 + years	445	445	306	408	128	151
**Total**	**1,779**	**1,779**	**1,429**	**1,700**	**561**	**804**
**Women**						
35-44 years	37	37	31	37	3	28
45-54 years	61	61	46	58	10	34
55-64 years	123	123	81	117	26	50
65-74 years	201	201	130	187	56	67
75 + years	376	376	163	336	78	70
**Total**	**798**	**798**	**451**	**735**	**173**	**249**
**TOTAL**						
35-44 years	154	154	132	152	17	113
45-54 years	320	320	266	314	75	186
55-64 years	609	609	493	592	194	283
65-74 years	673	673	520	633	242	250
75 + years	821	821	469	744	206	221
**Total**	**2,577**	**2,577**	**1,880**	**2,435**	**734**	**1,053**

NYHA: New York Heart Association functional class. EF: ejection fraction. GFR: glomerular filtration rate (ml/min/1.73 m^2).

Table 2. Average prevalence of the four main selection criteria of the DAPA-HF study in the REDINSCOR cohort in Spain.

Sex and age	1 NYHA II-IV	2 EF ≤ 40%	3 GFR > 30	1+2+3 Diabetes	1+2+3 No diabetes
	Prevalence				
**Men**					
35-44 years	100%	86.3%	98.3%	14.1%	85.9%
45-54 years	100%	84.9%	98.8%	30.0%	70.0%
55-64 years	100%	84.8%	97.7%	41.9%	58.1%
65-74 years	100%	82.6%	94.5%	50.4%	49.6%
75 + years	100%	68.8%	91.7%	45.9%	54.1%
**Total**	**100%**	**80.3%**	**95.6%**	**41.1%**	**58.9%**
**Women**					
35-44 years	100%	83.8%	100%	9.68%	90.3%
45-54 years	100%	75.4%	95.1%	22.7%	77.3%
55-64 years	100%	65.9%	95.1%	34.2%	65.8%
65-74 years	100%	64.7%	93.0%	45.5%	54.5%
75 + years	100%	43.4%	89.4%	52.7%	47.3%
**Total**	**100%**	**56.5%**	**92.1%**	**41.0%**	**59.0%**
**TOTAL**					
35-44 years	100%	85.7%	98.7%	13.1%	86.9%
45-54 years	100%	83.1%	98.1%	28.7%	71.3%
55-64 years	100%	81.0%	97.2%	40.7%	59.3%
65-74 years	100%	77.3%	94.1%	49.2%	50.8%
75 + years	100%	57.1%	90.6%	48.2%	51.8%
**Total**	**100%**	**73.0%**	**94.5%**	**41.1%**	**58.9%**

NYHA: New York Heart Association functional class. EF: ejection fraction. GFR: glomerular filtration rate (ml/min/1.73 m^2).

Table 3. Total number of patients with heart failure, ejection fraction ≤ 40%, New York Heart Association functional class II–IV, and glomerular filtration rate ≥ 30 mL/min/1.73 m^2 in Spain and its Autonomous Communities, according to prevalence data from the REDINSCOR registry (Tables S1 and S2).

		HF + EF ≤ 40% + NYHA = II–IV + GFR > 30			
	**Total**	**45-54**	**55-64**	**65-74**	**75+**
**Prevalence**		**98.1%**	**97.2%**	**94.1%**	**90.6%**
Andalucía	56,454	3,281	7,746	14,006	31,421
Aragón	11,140	507	1,277	2,477	6,880
Principado de Asturias	9,949	401	1,144	2,399	6,005
Illes Balears	7,342	475	1,028	1,883	3,957
Canarias	14,135	956	2,081	3,627	7,472
Cantabria	4,896	228	605	1,188	2,875
Castilla y León	23,680	921	2,563	5,017	15,179
Castilla-La Mancha	15,396	783	1,884	3,282	9,447
Catalunya	55,833	2,906	6,710	13,421	32,796
Comunitat Valenciana	37,313	1,956	4,664	9,246	21,446
Extremadura	8,659	406	1,070	1,900	5,283
Galicia	25,991	1,038	2,674	5,860	16,419

Comunidad de Madrid	46,403	2,627	5,854	11,092	26,830
Región de Murcia	9,231	574	1,253	2,238	5,167
Comunidad Foral de Navarra	4,993	251	602	1,176	2,965
País Vasco	18,868	850	2,218	4,504	11,296
La Rioja	2,582	120	305	583	1,574
Ciudad Autónoma de Ceuta	417	29	73	103	212
Ciudad Autónoma de Melilla	377	27	69	92	188
All Communities	353,658	18,337	43,817	84,094	207,409
95% CI	352,494- 354,825	18,074- 18,603	43,409- 44,228	83,528- 84,663	206,518 208,303

HF: heart failure. EF: ejection fraction. NYHA: New York Heart Association functional class. GFR: glomerular filtration rate (mL/min/1.73 m^2). CI: Confidence interval.
